# Return to sports after arthroscopic Bankart repair in teenage athletes: a retrospective cohort study

**DOI:** 10.1186/s12891-023-06145-y

**Published:** 2023-01-24

**Authors:** Yohei Harada, Yusuke Iwahori, Yukihiro Kajita, Ryosuke Takahashi, Shin Yokoya, Yasuhiko Sumimoto, Masataka Deie, Nobuo Adachi

**Affiliations:** 1 Department of Orthopaedic Surgery, Graduate School of Biomedical and Health Sciences, Hiroshima University, 1-2-3 Kasumi, Minami-Ku, Hiroshima City, Hiroshima, 734-8551 Japan; 2 Department of Orthopaedic Surgery, Aichi Medical University, School of Medicine, 1-1 Yazakokarimata, Nagakute, Aichi 480-1195 Japan; 3 Sports Medicine and Joint Center, Asahi Hospital, 2090 Shimoharacho Azamurahigashi, Kasugai, Aichi 486-0819 Japan; 4 Department of Orthopaedic Surgery, Ichinomiya Nishi Hospital, 1 Azahira, Kaimei, Ichinomiya City, Aichi 494-0001 Japan; 5 Department of Orthopaedic Surgery, Hiroshima Citizens Hospital, 7-33 Motomachi, Naka-Ku, Hiroshima City, Hiroshima, 730-8518 Japan

**Keywords:** Arthroscopic Bankart repair, Teenage athletes, Shoulder instability, Return to sports, Contact athletes, Overhead athletes

## Abstract

**Background:**

Anterior shoulder instability is frequent among young athletes. Surgical treatment for this injury aims to facilitate an early return to sports (RTS). However, the rate of recurrent instability after surgery is reportedly high among young patients, and it is unclear whether surgery ensures satisfactory RTS. The purpose of this study was to verify the clinical outcomes and RTS after arthroscopic Bankart repair in competitive teenage athletes without critical bone loss in the glenoid.

**Methods:**

We retrospectively reviewed competitive teenage athletes who underwent arthroscopic Bankart repair. Patients with large bony defects in the glenoid, larger than 20% of the healthy side, were excluded. Clinical outcomes, recurrent instability, the final level of RTS, and the time needed for RTS were analyzed.

**Results:**

In total, 50 patients with a mean follow-up period of 44.5 ± 19.6 (range, 24–85 months) months were included. The mean age at surgery was 16.8 ± 1.7 (range, 13–19 years) years. Two patients (4.0%) experienced recurrent instability. All patients returned to sports, 96% of patients participated competitively, and 76% achieved a complete return to the pre-injury level without any complaints. The time for RTS was 6.6 ± 2.7 months (range, 3–18 months), to competitions was 9.3 ± 4.0 (range, 6–24 months) months, and to complete return was 10.6 ± 4.3 (range, 8–24 months) months. The complete return rates varied by sports type, with 82% in contact athletes, 59% in dominant-hand overhead athletes, and 100% in other athletes (*P* = 0.026). Other preoperative factors or concomitant lesion such as bony Bankart, superior labrum tear, or humeral avulsion of glenohumeral ligament lesion did not affect the complete RTS.

**Conclusion:**

Arthroscopic Bankart repair is an effective surgical procedure for anterior shoulder instability, even among competitive teenage athletes. Sports type was the only factor associated with complete RTS after surgery.

## Background

Traumatic anterior shoulder instability is common among young athletes. The incidence rate of primary shoulder dislocation is the highest among teenagers in each generation [1, 2]. The natural course of primary anterior shoulder dislocation has been reported in approximately 90% of teenage patients who experienced recurrent instability during long-term follow-up [3, 4]. The primary dislocation may be treated conservatively at first; however, failure of the treatment leads to a persistent sensation of the shoulder loosening or further instability, which causes disability in sports activities or daily activities [3]. In such cases, surgical intervention is recommended.

Although the cause of anterior shoulder instability is multifactorial, injury of anteroinferior capsulolabral complex is one of the main causes [5], and Bankart lesions are the most frequent in young patients. Arthroscopic Bankart repair (ABR) is the most performed procedure, and good clinical results have been reported [6]. Especially, a case without obvious bony defects, which is known as an “on-track” case, is reportedly a good indication for ABR [7]. However, younger age is a risk factor of recurrence after arthroscopic stabilization [8, 9]. Furthermore, athletes demand not only shoulder stability but also complete return to sports (RTS) at the earliest after surgical intervention. Although the recurrence rate after ABR has been well reported [6], the rate of complete RTS or the duration needed for RTS in teenage athletes is not well known.

The purpose of this study was to evaluate the recurrence rate and RTS after ABR in teenage athletes who had no significant bony defects in the glenoid. Furthermore, we sought to identify preoperative and intraoperative factors that influenced RTS. We hypothesized that ABR would provide a good RTS, but complete recovery to sports activity and the time needed for RTS would depend on the type of sports.

## Methods

### Patients

This was a retrospective cohort study of teenage athletes who underwent ABR for traumatic anterior shoulder instability between January 2013 and January 2019 at a single institution by a single surgeon. Informed consent was obtained from all participants and their parents, and institutional review board approved this study (2020–008). All patients preoperatively underwent computed tomography (CT) of both shoulders, and the size of the bony defect of the glenoid was measured. The inclusion criteria were as follows: (1) age of 13–19 years at the time of surgery, (2) athletes who regularly played sports in the school team, local club, or work team at a competitive level; (3) athletes who aimed RTS after the surgery; (4) patients who underwent ABR with or without any additional intra-articular procedure such as bony Bankart repair, humeral avulsion of glenohumeral ligament (HAGL) repair, superior labrum anterior and posterior (SLAP) repair. We excluded patients who underwent remplissage procedure for large Hill–Sachs lesions [10], (5) patients with direct follow-up for more than one year after surgery, and indirect follow-up via telephone survey for more than two years after surgery. The exclusion criteria were as follows: (1) patients who decided to quit their sports before surgery, (2) patients with large bony defect of the glenoid, which is larger than 20% of the length of the healthy side [11]; in such cases, the modified Bristow procedure was performed in addition to ABR. The distinctive types of sport were divided into three groups according to Ide et al. [12]: contact sport, dominant overhead sport, and noncontact-nonoverhead sport. Contact sport includes sports in which bodies collide intensely with each other, such as rugby and soccer, as well as martial arts sports such as judo, wrestling, and boxing. The dominant overhead sport includes sports involving pitching such as baseball, and overhead sports using a racket such as a badminton, as well as sports in which overhead movements are repeated, such as volleyball, basketball, handball, and swimming. The noncontact-nonoverhead sport includes other sports and overhead sports in which the affected side is not dominant side.

### Surgical procedure

**Fig. 1:**
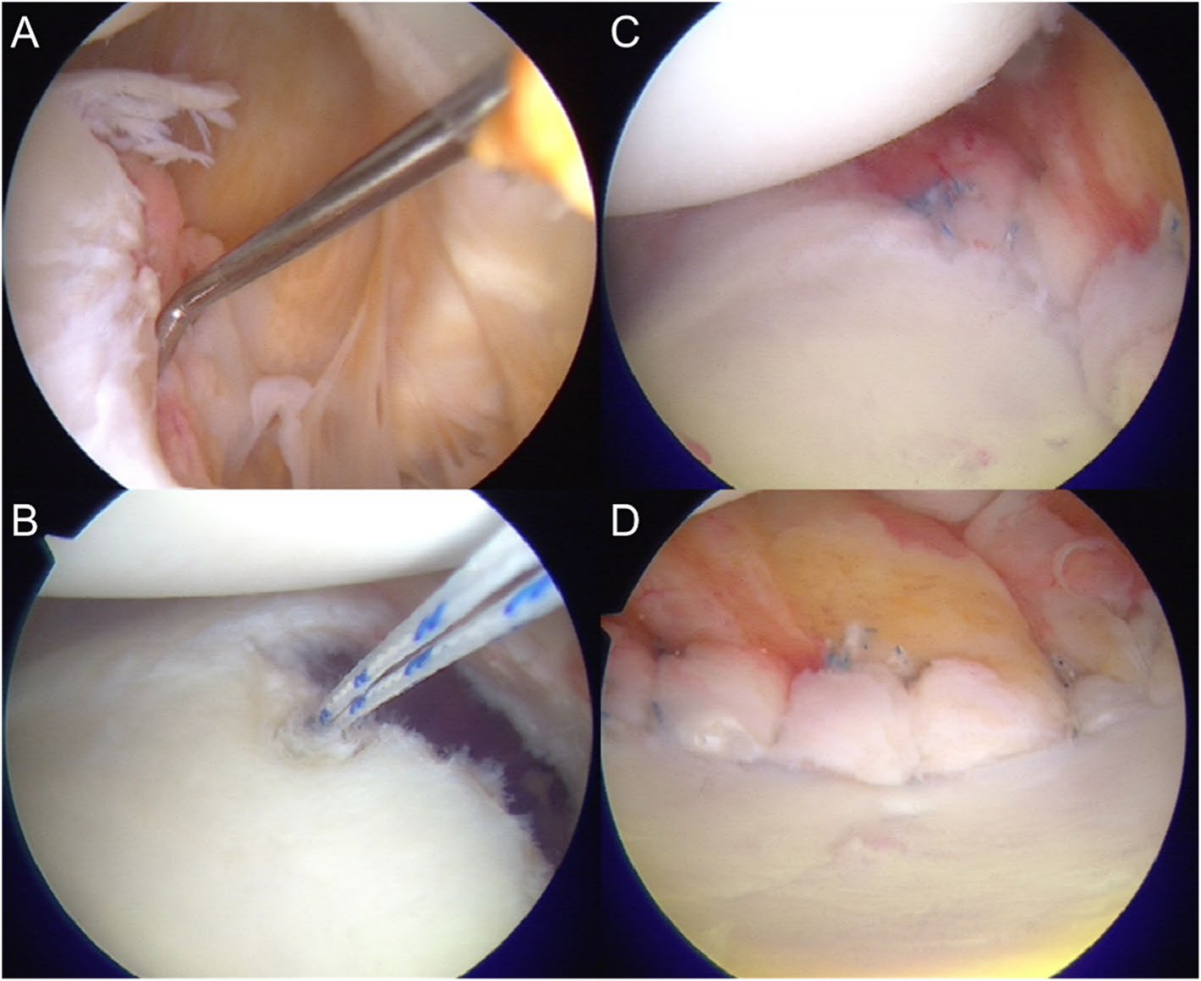
Intraoperative findings of arthroscopic Bankart repair of the left shoulder. **A** Detachment of anterior and inferior glenohumeral ligament labrum complex (IGLLC), viewing via the anterosuperior portal. **B** After mobilization of IGLLC, the first anchor at 6 o’clock position was inserted, viewing from the anterosuperior portal. **C** After the Bankart repair, viewing from the anterosuperior portal. **D** Four to seven anchors were inserted at the glenoid rim for Bankart repair, viewing from the posterior portal

**Fig. 2:**
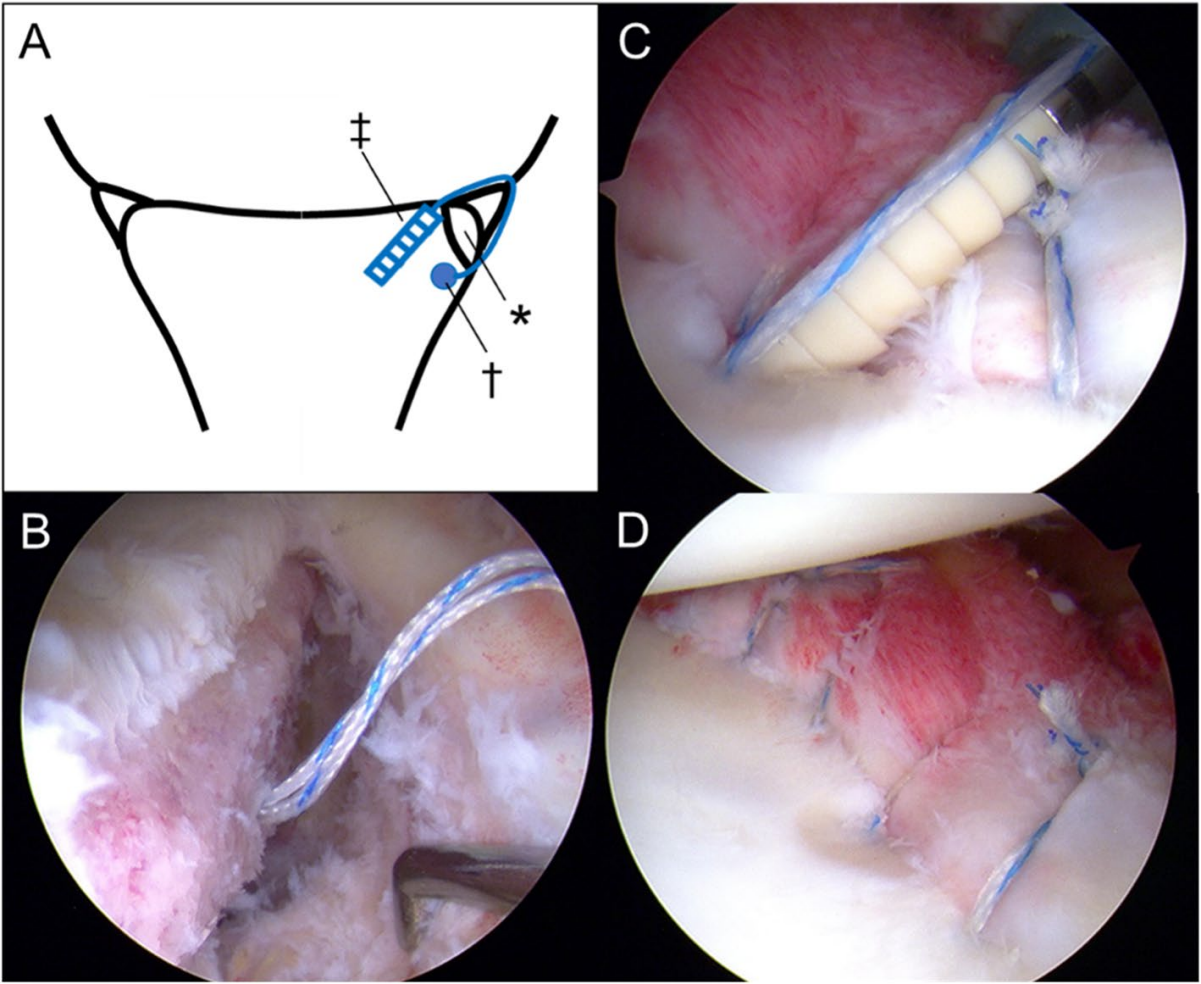
A schema and intraoperative findings of the case with bony Bankart lesion repaired using the double-row technique. **A** A schema of the technique. * indicates bony Bankart lesion, † represents medial anchor at the glenoid neck, ‡ shows push-lock anchor inserted at the glenoid rim. **B** A medial suture anchor was inserted at the glenoid neck after detachment of bony Bankart lesion together with inferior glenohumeral ligament labrum complex, viewing via the anterosuperior portal. **C** A push-lock anchor was inserted at the glenoid rim to fix the bony fragment with the glenohumeral ligament labrum complex, viewing the posterior portal. **D** After the bony Bankart repair, viewing from the anterosuperior portal

Under interscalene block and general anesthesia, patients were placed in the lateral decubitus position with the affected arm placed in 20° abduction using 2-kg inferior traction and 2–3-kg lateral traction. A standard posterior portal was made in addition to the anterior and anterosuperior portals into the rotator interval. The anterior portal was located 1 cm laterally and 1 cm inferiorly from the coracoid process to make it easier to place the anchor at 6 o’clock. After the diagnostic assessment of intra-articular pathology, the inferior glenohumeral ligament labrum complex (IGHLLC) was completely mobilized from the glenoid. This mobilization was performed beyond the 6 o’clock position of the glenoid to induce re-tension in the IGHLLC. The glenoid neck was decorticated, and 4 to 7 suture anchors (JuggerKnot Soft Anchor 1.4-mm, Biomet Sports Medicine, Warsaw, IN) were inserted at the anteroinferior glenoid rim. The first anchor was placed close to the 6 o’clock position, using a curved guide. Viewing through the anterosuperior portal, a suture passer was introduced from the posterior portal, and the suture was passed through the capsule immediately inferior to the first anchor. The viewing portal was changed to the posterior portal, and the suture was tied to grasp and pull up the IGHLLC. The following suture anchors were superiorly inserted at one o’clock intervals, and the same procedures were repeated two to five times (Fig. 1). If a large bony Bankart was present, the double-row technique was used. In this technique, one or two suture anchors were inserted at the glenoid neck at 3 to 5 o’clock, and the sutures were passed through the capsule and the bony fragment and fixed at the glenoid rim using a knotless anchor (2.4 mm PushLock anchor, Arthrex, Naples, FL) (Fig. 2). If HAGL, SLAP lesions, or posterior labral tears were concomitant, additional suture anchors were used for repair. Rotator interval closure was not applied in any of the cases.

### Postoperative management

All patients followed the same postoperative rehabilitation protocol under the supervision of one of the authors. A shoulder sling was used for four weeks. Isometric strengthening was initiated one day after surgery. After two weeks, self-assisted elevation training was initiated. Active elevation and abduction exercises were started at 3 and 4 weeks after the surgery, respectively. Sports activity was gradually started from 3 months, and overhead motion or contact play was permitted at 6 months. Return to play was permitted when the patient was pain-free and the range of motion and muscle strength had been restored to almost the same level as the healthy side.

### Clinical evaluation

The data on preoperative assessment, including age at surgery, sex, dominant side, number of preoperative instability episodes, the time from the first injury to surgery, and participating sports, were collected from medical records. Preoperative CT images were assessed and subcritical bone loss was defined as a bony defect of 13.5%–20% of the healthy glenoid [13]. Intraoperative findings such as complications with bony Bankart lesions, HAGL lesion, SLAP lesion, and the number of anchors for Bankart repair inserted into the glenoid rim were also recorded.

The range of shoulder motion (ROM), the Rowe score, Japan Shoulder Society Shoulder Instability Score (JSS-SIS) [14], and JSS Shoulder Sports Score (JSS-SSS) [15] were assessed before surgery and at the final visit. The ROM include the forward flexion, external rotation (ER) in adduction, internal rotation (IR) behind the back, and the external rotation and internal rotation in 90 degrees shoulder abduction and 90 degrees of elbow flexion (ER in abduction and IR in abduction). The JSS-SIS is a 100-point scoring system based on pain, function, range of motion, radiographic evaluation, and stability. The JSS-SSS is also a 100-point scoring system based on the player's ability, pain, muscle strength, and range of motion. Clinical improvement was also evaluated using minimal clinically important difference (MCID) for the Rowe score, defined as increasing more than 9.7 from the baseline [16]. The final status of RTS, recurrence of instability, and subjective scores such as the Oxford Shoulder Instability Score (OSIS) [17], American Shoulder and Elbow Surgeons (ASES) score [18], and Athletic Shoulder Outcome Scoring System (ASOSS) [19], except for the ROM item, were assessed at the final follow-up via telephone survey. The status of RTS was categorized as follows: (1) complete return: same as or superior to the preinjury level without any shoulder problems; (2) incomplete return to competition: same as preinjury level with any shoulder problems, or inferior to the preinjury level but participated in competitions; (3) unable to return to competition: participated in sports only in practice; and (4) unable to RTS.

### Statistical evaluation

All statistical analyses were performed using IBM SPSS software (version 22; IBM, Armonk, NY, USA). A preliminary test for normality was examined using a preliminary Shapiro–Wilk test. The outcomes before and after surgery was compared by Wilcoxon’s signed-rank test or paired t-test. To analyze the factors that influenced the complete RTS, preoperative and intraoperative factors were compared between patients who could achieve complete RTS and those who could not, and Mann–Whitney U test, Student’s t-test, chi-square test, and Fisher’s exact test were used for the comparison. Postoperative outcomes were also compared between the two groups. Postoperative outcomes were compared according to sports type using the Kruskal–Wallis test or one-way ANOVA.

## Results

### Patients

Fifty athletes were analyzed, and their demographic data are shown in Table 1. A total of 15 female and 35 male patients underwent surgery at a mean age of 16.8 ± 1.7 years (range, 13–19 years). Preoperative instability episodes were single in 10 patients and multiple in 40 patients. The affected arm side was the dominant side in 36 patients and the non-dominant side in 14 patients. The average duration from the first injury to surgery was 16.2 ± 12.9 months (range, 3–95 months). The mean follow-up period was 44.5 ± 19.6 months (range, 24–85 months). Seventeen patients were contact athletes, 22 were overhead athletes on their dominant side, and 11 were other athletes.

**Table 1:** Patient demographic data

Age	16.8 ± 1.7 years
Sex	
Female	15
Male	35
Affected arm side	
Dominant	36
Non-dominant	14
Number of preoperative instability episodes	
Single	10
Multiple	40
Time from the first injury to surgery	16.2 ± 12.9 months
Type of sports	
Contact	
Rugby	6
Soccer	7
Futsal	1
Wrestling	1
Judo	1
Boxing	1
Overhead on dominant	
Baseball	8
Softball	3
Handball	2
Volleyball	2
Basketball	2
Badminton	3
Tennis	1
Swimming	1
Noncontact-nonoverhead	
Non-dominant overhead	7
Skiing	1
Diving	1
Cheerleading	1
Dancing	1

### Intraoperative findings and complications

All patients had Bankart lesions, and 15 had bony fragments, 3 of whom had primary dislocations and 12 had recurrent instabilities. The average number of anchors used for Bankart repair at the glenoid rim was 5.9 ± 0.7 (range, 4–7). Twelve patients had SLAP lesions, and six had HAGL lesions, all of which underwent Bankart repair. There were no intraoperative or postoperative complications related to surgery.

### Clinical outcomes

The Rowe score, JSS-SIS, and JSS-SSS significantly improved from 40.7 ± 13.2 to 96.6 ± 9.8, 56.3 ± 9.0 to 96.7 ± 6.1, and 50.0 ± 15.4 to 93.7 ± 8.9, respectively, from the preoperative to the postoperative period. Forty-seven patients (94%) achieved MCID improvement of the Rowe score. The mean OSIS, ASES score, and ASOSS at the final follow-up were 47.5 ± 1.0, 99.4 ± 1.2, and 86.4 ± 6.4, respectively. Two patients (4%) experienced recurrent dislocation or subluxation.

### Return to sports

All patients returned to sports, but two patients could only participate in the practice. Ten patients could participate in competition at an inferior level than before the injury or pre-injury level with some complaints in their shoulders. Thirty-eight patients (76%) returned to sports at the same or higher level than before the injury, without any shoulder complaints. Among the athletes who did not reach the level of complete return, two complained of instability, two had an anxiety to play to their complete ability, two had slight pain, two had restriction of motion, and four had indefinite complaints such as discomfort or a strange feeling. The duration of return to play was 6.6 ± 2.7 months (range, 3–18 months), to competitions was 9.3 ± 4.0 months (range, 6–24 months) and to the level of complete return was 10.6 ± 4.3 months (range, 8–24 months).

### Factors affecting the complete return to sports

Age, sex, dominant side, number of preoperative instability episodes, the time from the first episode to surgery, and subcritical bone loss were not risk factors for failure of complete RTS. The type of sports was associated with failure of complete RTS. Regarding intraoperative findings, concomitant lesions and the number of anchors used for Bankart repair were not associated with the failure. Final ROM did not differ between the athletes with complete RTS and incomplete RTS. (Table 2).

**Table 2:** Risk factors affecting the complete return to play

	Complete return (*N* = 38)	Incomplete return (*N* = 12)	*P*
Age (years)	16.8 ± 1.8	16.6 ± 1.5	0.68
Sex			0.31
Female	10	5	
Male	28	7	
Number preoperative instability episodes			0.74
Single	8	2	
Multiple	30	10	
Time from first injury to surgery (months)	16.6 ± 12.6	15.1 ± 14.8	0.55
Type of sports			0.026
Contact	14	3	
Overhead on dominant	13	9	
Noncontact-nonoverhead	11	0	
Subcritical bone loss	9	2	1.00
Intraoperative concomitant lesion			
Bony Bankart	12	3	1.00
SLAP	8	4	0.45
HAGL	4	2	0.62
Number of anchors for Bankart Repair at the glenoid rim	5.8 ± 0.7	6.0 ± 0.9	0.56
ROM at final follow up			
Flexion (°)	181 ± 6	180 ± 3	0.95
ER in adduction (°)	72 ± 10	70 ± 10	0.56
IR behind the back	T6 ± 2	T7 ± 1	0.26
ER in abduction (°)	93 ± 8	93 ± 5	0.71
IR in abduction (°)	24 ± 8	25 ± 11	0.90

*SLAP* Superior labrum anterior and posterior, *HAGL* Humeral avulsion of glenohumeral ligament, *ROM* Range of motion, *ER* External rotation, *IR* Internal rotation

### Clinical outcomes by sports type

Although statistical tests could not be performed due to the small number of cases with recurrence, there was no difference in the recurrence rate of instability between sport groups. The rate of complete RTS differed by sports type, which was 82% in contact athletes, 59% in overhead athletes on the dominant hand side, and 100% in noncontact-nonoverhead athletes. The duration of return to complete RTS was longer in overhead athletes. Postoperative Rowe score and OSIS were not significantly different between the groups, but JSS-SIS, JSS-SSS, ASES, and ASOSS scores were lower in overhead athletes. (Table 3).

**Table 3:** Clinical outcomes by sports type

	Contact athletes (*N* = 17)	Dominant-hand overhead athletes (*N* = 22)	Noncontact-nonoverhead athletes (*N* = 11)	*P*
Recurrence of instability	1	0	1	-
Complete RTS	14 (82.3%)	13 (59.1%)	11 (100%)	0.026
Duration for RTS (months)	5.8 ± 2.8	6.5 ± 1.8	7.8 ± 3.9	0.20
Duration for RTS in competition (months)	7.8 ± 3.9	10.4 ± 4.1	9.7 ± 3.6	0.10
Duration for complete RTS (months)	8.3 ± 3.8	13.0 ± 3.9	10.8 ± 4.1	0.021
Postoperative score				
JSS-SIS (/100)	99.3 ± 1.5	94.8 ± 6.4	96.2 ± 8.8	0.011
JSS-SSS (/100)	96.6 ± 4.6	89.1 ± 11.2	98.1 ± 4.5	0.011
The Rowe score (/100)	97.6 ± 9.7	96.6 ± 6.8	94.5 ± 15.7	0.11
OSIS (/48)	47.9 ± 0.5	47.4 ± 1.0	47.3 ± 1.6	0.10
ASES (/100)	99.9 ± 0.2	99.0 ± 1.6	99.5 ± 1.1	0.023
ASOSS (/90)	86.9 ± 4.7	84.5 ± 8.4	89.4 ± 1.3	0.040

*RTS* Return to sports, *JSS-SIS* JSS shoulder instability score, *JSS-SSS* JSS shoulder instability score, *OSIS* Oxford shoulder instability score, *ASES* American shoulder and elbow surgeons score, *ASOSS* Athletic shoulder outcome scoring system (except ROM item)

## Discussion

The findings of this study suggest that ABR is effective even in teenage athletes with anterior shoulder instability in the absence of critical glenoid bone loss. In total, 96% of patients restored stability after ABR, and 76% achieved complete RTS without any shoulder complaints. ABR also provided excellent clinical scores, such as OSIS, ASES, and ASOSS scores.

The treatment goals for anterior shoulder instability in young athletes are to regain shoulder stability and reestablish normal shoulder biomechanics and complete RTS without any symptoms. After the first-time traumatic anterior shoulder instability, immobilization in external rotation has been reported to have a lower rate of recurrent instability and a higher RTS rate than immobilization in internal rotation [20, 21]. However, in general, conservative treatment of anterior traumatic instability in young athletes has been reported to have a high recurrence rate. Robinson et al. [3]. reported that patients of 15 to 20 years of age showed a redislocation rate of 52.0% in one year, 72.6% in two years, and 86.6% in five years after the first dislocation. Hovelius et al. [20] also reported recurrent instability in 62.8% of athletes of 12–22 years of age at 5 years of follow-up. Furthermore, a 10-year prospective study by Hovelius et al. [21] reported that approximately 40% of patients of 12–19 years of age eventually required surgical stabilization. Zaremski et al. [22] reported that the primary non-operative group was more prone to have recurrence with an odds ratio of 13.41, compared to the primary operation group in a meta-analysis of adolescent athletes. They also concluded that surgical stabilization is effective for RTS, with a rate of 95.3% for primary operative patients and 44.4% for primary non-operative patients.

Regarding the rate of recurrent instability after ABR, it has been reported that it is higher in younger patients. Porcellini et al. [8] reported that the rate of redislocation was 13.3% among patients ≤ 22 years of age and 6.3% among older patients. Torrance et al. [9] reported a higher recurrence rate of 51% in contact athletes of ≤ 18 years of age, compared with a 12% recurrence rate in the control series of 25-year-old athletes. Furthermore, they reported that even in adolescent athletes, the risk of recurrence was 2.2 times higher in athletes < 16 years of age than in those > 16 years of age. Adolescent athletes have also reported different recurrence rates depending on the surgical technique. According to a review of surgical stabilization of pediatric anterior shoulder instability [23], the recurrence rate was 24% in ABR, 12% in open Bankart repair, 6% in modified Bristow, and 8% in Latarjet procedure. Considering this high recurrence rate in pediatric athletes, the recurrence rate in this study was low at 7.2%. However, the recurrence rate depends on the type of sports, and high recurrence rates have been reported in contact and collision sports [24, 25], especially in rugby and American football [26, 27]. Our study included only six rugby players in these high-risk sports. Other factors that influence instability include surgical technique and tensioning of the IGHLLC, which is vital in ABR [28, 29]; further, an insufficient number of anchors is considered a risk factor of recurrence [30, 37, 38]. In the surgical technique applied in this study, the IGHLLC was detached inferiorly beyond 6 o'clock, the complex was re-tensioned, and at least four anchors were inserted into the glenoid rim for repair. Furthermore, the current study did not include patients with sizeable bony loss of the glenoid, which is at risk of failure with Bankart repair alone, and we believe this is another factor contributing to the low recurrence rate in our study.

ABR was reported to have the highest rate of RTS in all age groups compared to other stabilization surgeries such as open Bankart, open Latarjet, and arthroscopic Latarjet [31]. In a review of teenage RTS after surgical stabilization, return at any level was reported as 95%, and the pre-injury level was 77% [23]. The present study showed similar results, and the return at any level was 100%, with the return at the pre-injury level of 76%. However, the RTS is expected to depend on the sports type, and the only factor affecting the complete RTS in this study was the type of sports, and the overhead athletes showed the lowest rate. Ide et al. [12] also reported the complete return rate after ABR by sports, and overhead athletes had the lowest rate of 68%, compared to 86% in contact athletes and 100% in noncontact-nonoverhead athletes. In overhead athletes, RTS without any symptoms after shoulder stabilization surgery is thought to be difficult because they require a range of motion and stability. Inadequate improvement of external rotation in abduction was reportedly associated with incomplete RTS after ABR in overhead athletes [32]. The duration of complete RTS tended to be longer in overhead athletes in our study, which may be related to the fact that sufficient recovery of range of motion is necessary for complete RTS in overhead athletes.

In this study, complete RTS in contact athletes was also low (82%) compared to noncontact-nonoverhead athletes. According to past reports on outcomes after ABR in adolescent contact-collision athletes, recurrence of instability was 10.3%–51% [9, 33, 39], and RTS for the pre-injury level was 61%–78.1% [33, 39]. Nixon et al. [33] reported the primary arthroscopic shoulder stabilization in adolescents playing contact sports and that patients who could not return to the same level of sports were more prone to have recurrent instability. In our study, one contact athlete with sustained recurrent instability could not achieve complete RTS. Adolescent and competitive contact athletes have been reported to have a high failure risk after ABR [34], and a more aggressive indication for the Bristow-Latarjet procedure may be considered in this challenging population. The Bristow-Latarjet is generally used in patients with large glenoid bone defects and Hill-Sachs lesion involvement [7]. There is no consensus on the indications and contraindications of each technique in adolescent contact athletes with “on track” lesions, even if they have a high recurrence rate.

We acknowledge that there are several limitations to this study. This was a retrospective study, and there was no comparison between adult athletes and other surgical techniques. The sample size was small, and the number of athletes in each sport was heterogeneous; therefore, it was impossible to examine the recurrence risk and RTS for each sport. Moreover, although we have classified sports into three groups, there are various ways to classify sports, which makes it difficult to compare our results to other studies. The small sample size also precluded the possibility that the heterogeneity of intraoperative concomitant or little-to-moderate bone loss may have influenced the results. Despite these limitations, this study presented the results of ABR in a homogeneous group of competitive teenage athletes without large glenoid bone loss.

## Conclusion

ABR is an effective surgical procedure for anterior shoulder instability, even among competitive teenage athletes in the absence of critical bone loss of the glenoid. The recurrence rate was 4% with an average follow-up of 45 months, and 76% of patients returned to sports at the pre-injury level without any shoulder complaint at a mean of 10.6 months. Sports type was the only factor associated with complete RTS after surgery.

## Data Availability

The datasets used and/or analysed during the current study are available from the corresponding author on reasonable request.

## Abbreviations

ABR: Arthroscopic Bankart repair
RTS: Return to sports
CT: Computed tomography
HAGL: Humeral avulsion of glenohumeral ligament
SLAP: Superior labrum anterior and posterior
IGHLLC: Inferior glenohumeral ligament labrum complex
JSS-SIS: Japan shoulder society shoulder instability score
JSS-SSS: Japan shoulder society shoulder sports score
ER: External rotation
IR: Internal rotation
MCID: Minimal clinically important difference
OSIS: Oxford shoulder instability score
ASES: American shoulder and elbow surgeons
ASOSS: Athletic shoulder outcome scoring system
